# The association of elevated serum lipocalin 2 levels with diabetic peripheral neuropathy in type 2 diabetes

**DOI:** 10.1530/EC-21-0290

**Published:** 2021-10-12

**Authors:** Xi Zhang, Xiurong Shen, Wan Zhou, Mengyun Xu, Yan Xing, Jianping Weng, Shandong Ye, Suowen Xu, Zhi Zhang, Wei Wang

**Affiliations:** 1 Department of Endocrinology and Laboratory for Diabetes, The First Affiliated Hospital of USTC, Division of Life Sciences and Medicine, University of Science and Technology of China, Hefei, Anhui, People’s Republic of China; 2 Graduate School, Wannan Medical College, Wuhu, Anhui, People’s Republic of China; 3 Institute of Endocrine and Metabolic Diseases, University of Science and Technology of China, Hefei, Anhui, People’s Republic of China; 4 Hefei National Laboratory for Physical Sciences at the Microscale, Division of Life Sciences and Medicine, University of Science and Technology of China, Hefei, Anhui, People’s Republic of China

**Keywords:** diabetic peripheral neuropathy, diabetic complication, lipocalin 2, type 2 diabetes

## Abstract

A variety of studies have demonstrated the role of lipocalin 2 (LCN2) in both diabetes and neurological disorders. Nevertheless, the relationship between LCN2 and diabetic peripheral neuropathy (DPN) needs to be elucidated in humans. Therefore, this study aimed to investigate the association of LCN2 with DPN in type 2 diabetes (T2D). A total of 207 participants with T2D and 40 participants with normal glucose tolerance (NGT) were included in this study. All participants were classified into DPN group and non-DPN (NDPN) group based on the Toronto Clinical Neuropathy Scoring (TCNS). Demographic and biochemical parameters were measured. Serum LCN2 levels were determined using an ELISA technique. Serum LCN2 levels in NGT group were lower than those in either DPN group (*P* = 0.000) or NDPN group (*P* = 0.043), while serum LCN2 levels in DPN group were higher than NDPN group (*P* = 0.001). Moreover, serum LCN2 levels positively correlated to TCNS scores, which reflects neuropathy severity (*r* = 0.438, *P* = 0.000). Multivariate stepwise regression analysis showed that BMI, triglycerides, and diastolic pressure were independently associated with serum LCN2 in DPN. Additionally, logistic regression analysis demonstrated that LCN2 (odds ratio (OR) = 1.009) and diabetes duration (OR = 1.058) were independently associated with the occurrence of DPN in T2D. Our report reveals the association of serum LCN2 with DPN in T2D. LCN2 might be used to evaluate DPN severity and serve a role in the pathogenesis of DPN.

## Introduction

Type 2 diabetes (T2D), the most common form of diabetes, has fallen into the leading causes of disability and death worldwide. The harms of T2D arise from its complications such as neuropathy, nephropathy, retinopathy, and cardiovascular disease. Diabetic peripheral neuropathy (DPN), the most prevalent and troublesome complication of T2D, affects up to 50% individuals (1, 2). DPN, commonly with symptoms of pain, paresthesia, or numbness, leads to lower quality of life, increased morbidity, and huge economic burdens. A variety of factors related to the pathophysiological processes of T2D including hyperglycemia, the formation of intracellular advanced glycation end products, oxidative stress, mitochondrial dysfunction, and inflammatory cascades have been demonstrated to be implicated in the development and progression of DPN (3). However, the precise mechanisms underlying the development and progression of DPN remain elusive.

Lipocalin 2 (LCN2), also known as 24p3 or neutrophil gelatinase-associated lipocalin, is a secreted glycoprotein in response to numerous physiological and pathological stimuli (4). A variety of studies have demonstrated that LCN2 is broadly expressed in many tissues such as the adipose tissue, liver, kidneys, lungs, and the brain and serves roles in both metabolic and neurological disorders. Our previous study and other colleagues’ studies indicate that serum LCN2 levels are significantly higher in diabetes and diabetic complications such as diabetic retinopathy, diabetic nephropathy, and cardiovascular diseases (5, 6, 7, 8, 9). For neurological diseases, the role of LCN2 has been implicated in diabetic encephalopathy and some other neurological diseases in animal models with metabolic disturbance (10, 11). Most recently, a report by Bhusal *et al.* indicated that glial-derived LCN2 played an important role in the pathogenesis of DPN via PDK2-lactic acid axis in the dorsal root ganglion (DRG) in mice model (12). Nevertheless, the relationship between LCN2 and DPN in humans remains unclear. Therefore, we conducted a study to investigate the association between serum LCN2 and DPN in individuals with T2D.

## Materials and methods

### Participants

A total of 207 participants with T2D from the Department of Endocrinology, the First Affiliated Hospital of University of Science and Technology of China (USTC) were included in this study from September 2018 to February 2021. As the control, 40 participants with normal glucose tolerance (NGT) from the Health Examination Center, the First Affiliated Hospital of USTC, were recruited from October 2018 to May 2019. NGT and T2D were diagnosed according to the 1999 World Health Organization criteria. All participants with T2D were classified into DPN group and non-DPN (NDPN) group. The diagnosis of DPN was based on the Toronto Clinical Neuropathy Scoring (TCNS) (13). A total of 107 participants with TCNS scores ≥6 were assigned to the DPN group, and 100 participants with scores <6 were assigned to the NDPN group. The clinical parameters including sex, age, and BMI were matched in the two groups. The exclusion criteria were as follows: (a) type 1 diabetes, gestational diabetes, specific types of diabetes, or acute complications of diabetes; (b) neuropathy caused by other diseases or drugs; (c) severe arteriovenous vascular disease (e.g. venous embolism, lymphangitis); (d) neurotoxicity caused by drugs, especially chemotherapeutic drugs, and nerve damage caused by metabolic poisons caused by renal insufficiency; (e) chronic kidney disease ≥stage 3b; and (f) any amputation other than involving the toes or fingers.

The study was approved by the Ethics Committee of the First Affiliated Hospital of USTC and complied with the Declaration of Helsinki. Informed consents were obtained from all participants before inclusion in this study. This study was registered on the Chinese Clinical Trial Registry (ChiCTR2100046905).

### Data collection and demographic measurement

All participants were questioned and physical examination was done by trained doctors and nurses to obtain the information on age, sex, weight, height, blood pressure (BP), illness, and medical therapy history. The BMI was calculated as the weight/height^2^. BP was tested in triplicate after at least 30 min of rest, and the average of three recordings was recorded.

### Laboratory measurements

Venous blood samples were collected from all participants after an overnight fast of 10–12 h. Fasting blood glucose (FBG), total cholesterol (TC), triglycerides (TG), creatinine (in urine or serum) were assayed by an automatic biochemistry analyzer (7600-020 Chemical Analyzer, Hitachi, Japan). Hemoglobin A1c (HbA1c) was measured by affinity chromatography with an HbA1c radiometer (Bio-Rad Laboratory Inc.). Urinary albumin was assayed using immune turbidimetry kits purchased from Northern Biotechnology Research Institute (Beijing, China). Urine albumin creatinine ratio (UACR) was calculated as the urine albumin/urine creatinine. Fasting C-peptide (FCP) was assayed by electrochemiluminescence immunoassay (Roche Diagnostics GmbH). The estimated glomerular filtration rate was calculated by the Chronic Kidney Disease Epidemiology Collaboration equation.

Serum LCN2 levels were assayed using an ELISA kit (Shanghai Xitang Biotechnology Co. Ltd, Shanghai, China). Procedures were according to the manual instructions by kit provider.

### Assessment of DPN

DPN was diagnosed using TCNS based on neuropathic symptoms, signs, and the presence of abnormal nerve conduction (13). All participants underwent electromyogram tests to evaluate the median nerve motor and sensory branches, the motor and sensory branches of the ulnar nerve, the motor and sensory branches of the radial nerve, and the tibial nerve and the peroneal nerve. The TCNS, a validated and reliable scale, has been used to grade DPN severity (14). The clinical neuropathy score ranges from a minimum of 0 to a maximum of 19 points. Six points are derived from symptoms, eight from lower-limb reflexes, and five from sensory examination distally at the toes. A higher score indicates more severe disability.

### Statistical analysis

Statistical analyses were conducted using SPSS software (version 20.0, SPSS Inc). Continuous variables with normal distribution were expressed as means ± s.d., skewed variables as medians with interquartile ranges, and categorical variables as frequencies. All variables were tested for normality using Kolmogorov–Smirnov test. Skewed distributed variables including TG and UACR were logarithm transformed to normality for further analyses. To compare the differences between two groups, independent *t-*test was performed for the normally distributed variables, and *Χ^2^*-test for categorical variables. To compare the differences in the three groups, ANOVA was used followed by Bonferroni method. The correlation between LCN2 and TCNS score was performed by Spearman’s correlation analysis. The Pearson’s correlation analysis was used to examine the correlation between serum LCN2 and clinical parameters in DPN. Multivariate stepwise regression analysis was further performed to assess the association between serum LCN2 and clinical parameters after adjusting for potential confounders. Multiple logistic regression analysis was performed using the occurrence of DPN as a dependent variable. The confounders included the variables that had been reported to be associated with DPN. The *P* value less than 0.05 was considered to be statistically significant.

## Results

### Characteristics of the participants

As shown in Table 1, there were no statistically significant differences in sex, age, and BMI between both DPN group and NDPN group (all *P* > 0.05). When compared with the NDPN group, patients in DPN group had longer diabetes durations (*P* = 0.008). For the other variables that might affect serum LCN2 levels including HbA1c, FBG, FCP, TC, TG, UACR, eGFR, and BP, no statistically significant differences were observed between the two groups (all *P* > 0.05).

**Table 1:** General characteristics of the participants.

	NDPN group (*n* = 100)	DPN group (*n* = 107)	*P*
Age (years)	54.45 ± 11.89	57.18 ± 13.04	0.118
Male, *n* (%)	61 (61.00)	71 (66.36)	0.381
Diabetes duration (years)	7.49 ± 6.77	10.14 ± 7.29	0.008^a^
BMI (kg/m^2^)	24.34 ± 3.67	24.17 ± 3.16	0.713
FBG (mmol/L)	8.40 ± 3.33	8.86 ± 3.05	0.303
HbA_1_c (%)	8.76 ± 2.17	9.04 ± 2.16	0.344
FCP (nmol/L)	0.35 ± 0.22	0.32 ± 0.19	0.426
TC (mmol/L)	4.20 ± 0.84	4.31 ± 0.95	0.366
TG^b^ (mmol/L)	1.47 (1.00–2.16)	1.53 (1.00–2.43)	0.506
SBP(mmHg)	129.45 ± 16.03	130.26 ± 17.79	0.731
DBP(mmHg)	82.99 ± 8.91	81.15 ± 9.64	0.156
UACR^b^ (mg/g)	14.85 (9.80–26.25)	17.00 (10.54–33.13)	0.285
eGFR (mL/min/1.73 m^2^)	109.87 ± 18.95	108.13 ± 17.20	0.490

Continues variables are expressed as mean ± s.d., median (25th–75th percentile), or as *n* (%).

^a^*P* < 0.01. ^b^Logarithm transformations were carried out before analysis.

DBP, diastolic blood pressure; DPN, diabetic peripheral neuropathy; eGFR, estimated glomerular filtration rate; FBG, fasting blood glucose; FCP, fasting C-peptide; HbA_1_c, hemoglobin A1c; NDPN, non-diabetic peripheral neuropathy; SBP, systolic blood pressure; TG, triglyceride; TC, total cholesterol; UACR, urine albumin creatinine ratio.

### Serum LCN2 levels and DPN

**Figure 1:**
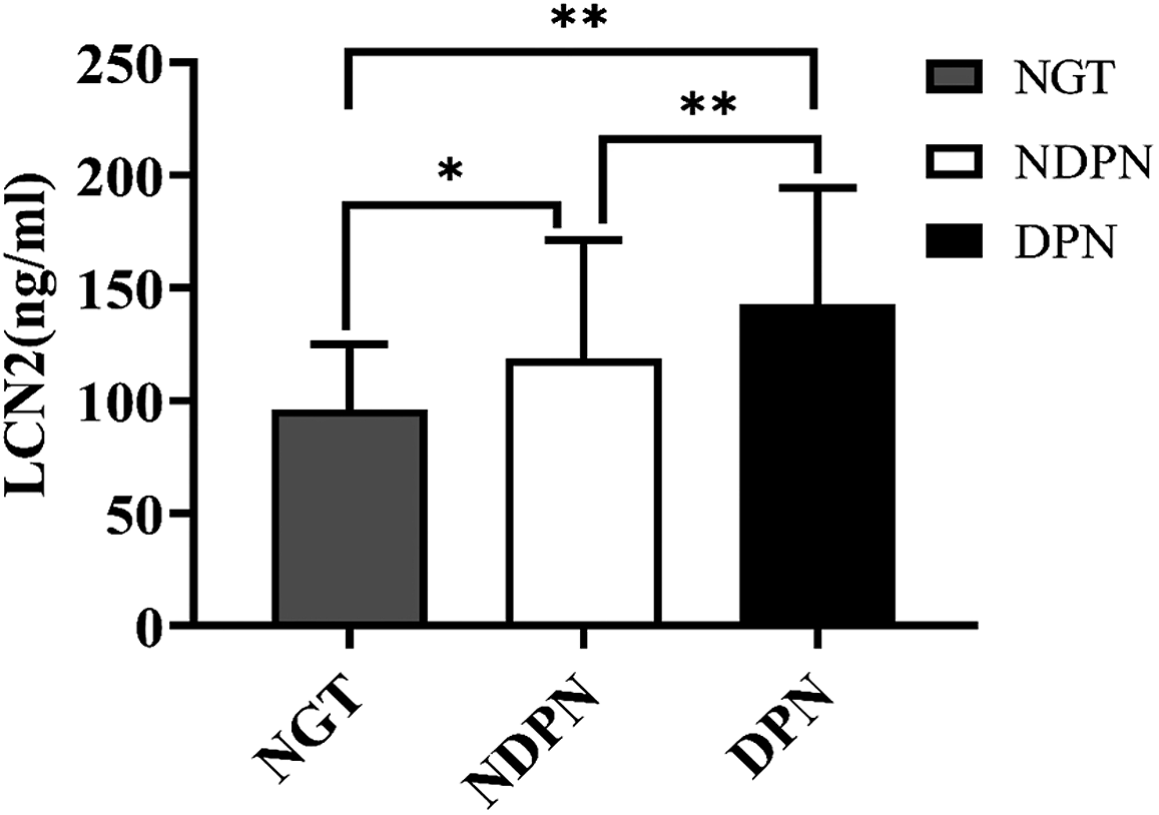
Comparison of serum LCN2 levels among NGT, NDPN, and DPN groups. DPN, diabetic peripheral neuropathy; LCN2, lipocalin-2; NDPN, non-diabetic peripheral neuropathy; NGT, normal glucose tolerance. *P < 0.05, **P < 0.001.

There were significant differences in serum LCN2 levels among NGT group (96.191 ± 28.322 ng/mL), DPN group (142.851 ± 51.195 ng/mL), and NDPN group (118.789 ± 52.042 ng/mL; *F* = 14.864, *P* = 0.000; Fig. 1). The serum LCN2 levels in NGT group were significantly lower than either DPN group or NDPN group, while LCN2 levels in DPN group were significantly higher than NDPN group (NGT vs DPN group, *P* = 0.000; NGT vs NDPN group, *P* = 0.043; NDPN vs DPN group, *P* = 0.001). The age, sex, and BMI in the three groups were matched (all *P* > 0.05).

**Figure 2:**
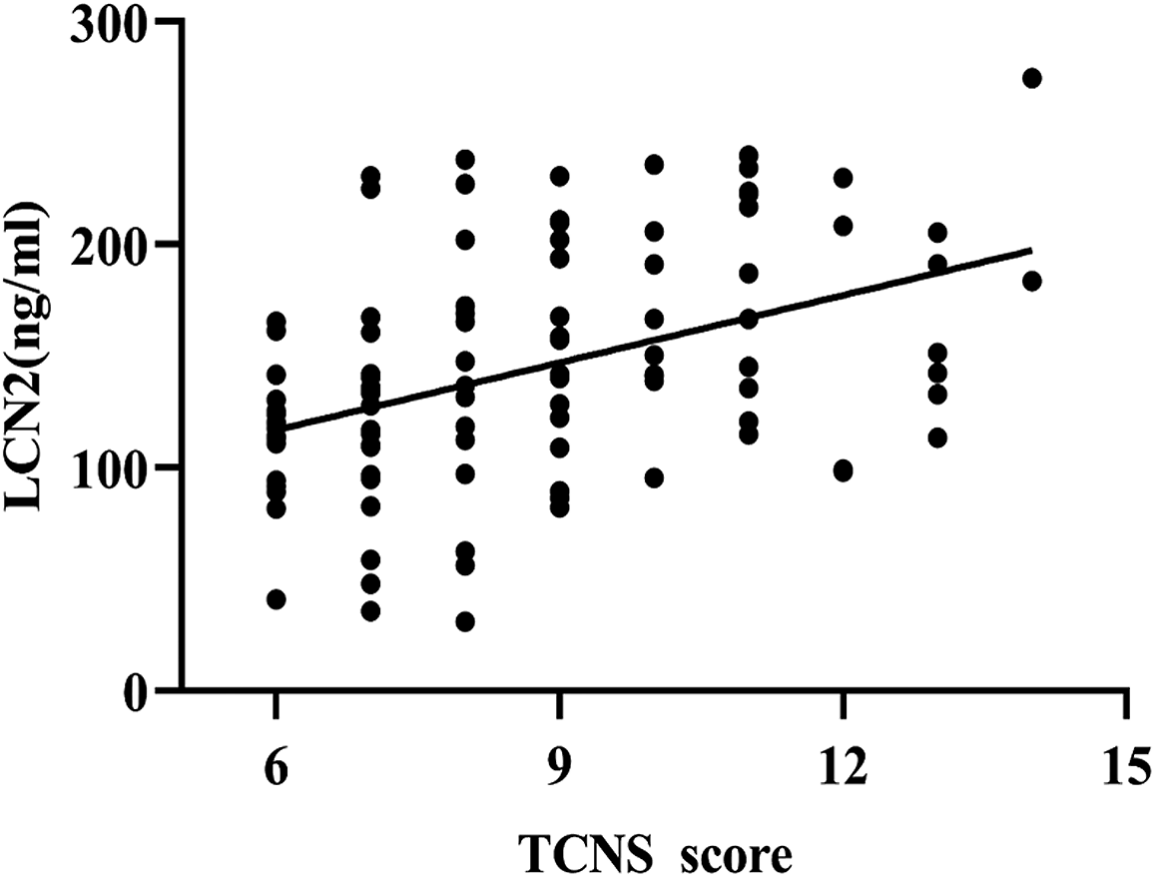
Positive correlation between TCNS score and serum LCN2 levels in DPN. DPN, diabetic peripheral neuropathy; LCN2, lipocalin 2; TCNS, Toronto Clinical Neuropathy Scoring.

Moreover, in participants with T2D, Spearman’s analysis showed that serum LCN2 level positively correlated to TCNS score, which reflects neuropathy severity (*r* = 0.438, *P* = 0.000; Fig. 2).

### The association of serum LCN2 with clinical parameters in DPN

To examine the association of clinical parameters with serum LCN2, Pearson’s correlation analysis was performed in DPN group. Serum LCN2 was shown to be positively correlated to BMI (*r* = 0.289, *P* = 0.003) and TG (*r* = 0.217, *P* = 0.024), respectively, and negatively correlated to DBP (*r* = −0.204, *P* = 0.035; Table 2). No significant correlations were observed between serum LCN2 and age, diabetes duration, HbA1c, FBG, TC, UACR, and SBP (all *P* > 0.05).

**Table 2:** Pearson’s correlation analysis of variables with serum LCN2 in DPN.

Variable	*r*	*P*
Age	−0.042	0.669
Diabetes duration	0.044	0.655
BMI	0.289	**0.003**
HbA_1_c	−0.091	0.350
FBG	−0.023	0.812
SBP	−0.146	0.133
DBP	−0.204	**0.035**
TG	0.217	**0.024**
TC	0.013	0.896
UACR	0.013	0.893

Significant differences are indicated in bold.

DBP, diastolic blood pressure; DPN, diabetic peripheral neuropathy; FBG, fasting blood glucose; HbA1c, glycosylated hemoglobin A1c; LCN2, lipocalin 2; SBP, systolic blood pressure; TG, triglyceride; TC, total cholesterol; UACR, urine albumin creatinine ratio.

To further determine independent clinical parameters affecting serum LCN2, multivariate stepwise regression analysis was performed using sex, age, BMI, HbA_1_c, UACR, eGFR, TG, and DBP as the independent variables. As shown in Table 3, sex (*β* = 21.933, *P* = 0.022), BMI (*β* = 4.666, *P* = 0.002), TG (*β* = 37.390, *P* = 0.022), and DBP (*β* = −1.835, *P*= 0.000) were shown to be independently associated with serum LCN2 levels in DPN.

**Table 3:** Multivariate stepwise regression analysis of serum LCN2 in DPN.

Independent variables	Dependent variable: serum LCN2			*P*
	*β*	SE	Standard *β*	
Sex	21.933	9.425	0.202	0.022
BMI	4.666	1.505	0.286	0.002
TG	37.390	16.116	0.215	0.022
DBP	-1.835	0.485	−0.344	0.000

DBP, diastolic blood pressure; DPN, diabetic peripheral neuropathy; LCN2, lipocalin 2; TG, triglyceride.

### Multiple logistic regression analysis

Multiple logistic regression analysis was performed using the occurrence of DPN as a dependent variable in all participants with T2D. As shown in Table 4, the independent factors for DPN were LCN2 (odds ratio (OR) = 1.009) and diabetes duration (OR = 1.058), respectively. Other variables were excluded in this model including sex, age, BMI, HbA1c, and UACR.

**Table 4:** Logistic regression analysis.

Independent variables	Dependent variable: occurrence of DPN				
	*β*	SE	*P*	OR	95% CI
LCN2	0.009	0.003	0.002	1.009	1.003–1.015
Diabetes duration	0.057	0.026	0.030	1.058	1.006–1.116

DPN, diabetic peripheral neuropathy; LCN2, lipocalin 2.

## Discussion

The mechanisms underlying the pathogenesis of DPN have been attributed to impaired glucose metabolism and dyslipidemia (15). The dysfunctions of metabolic pathways characterized by hyperglycemia and dyslipidemia cause an imbalance of the mitochondrial redox state and inflammatory processes, thereby leading to neuronal and glial cell injuries, which are accepted as crucial mechanisms of the pathogenesis of DPN (3). Increasing evidence has demonstrated the roles of LCN2 in modulating the activities of glial cells, recruiting immune cells, and amplifying neuroinflammation, consequently resulting in neuronal demyelination and apoptosis (4). Besides, as an iron-binding protein, LCN2-mediated oxidative stress promotes neuronal injury (10, 16). The LCN2 levels are known to be elevated in circulation in diabetes (5, 6, 7, 8, 9, 17). Consistently, increased LCN2 expressions have also been described in the brain regions in both ob/ob mice and mice fed high-fat diets, the two classical models with metabolic disorders characterized by obesity, hyperglycemia, dyslipidemia, systemic inflammation, and neuroinflammation (10, 18, 19). LCN2-related reactive oxygen species genes, which contribute to neurodegeneration, have been shown to be differentially expressed in the hippocampus of WT and *ob*/*ob* mice (10). Notably, the involvement of LCN2 has recently been implied in the neurological disorders from the studies in diabetic rodent models (10, 11). Bhusal *et al.* found that the expression of LCN2 in the hippocampus was increased in streptozotocin-induced diabetic mice models (11, 20). Deletion of *Lcn2* gene ameliorated diabetes-induced reactive gliosis and expression of pro-inflammatory cytokines in the hippocampus, subsequently decreasing neuronal loss in the hippocampus. Moreover, diabetes-associated cognitive deficits were improved in *Lcn2* knockout mice compared to WT mice in diabetic conditions.

Preclinical studies strongly suggest the presumable role of LCN2 in the pathogenesis of DPN in humans. This study herein first reported the association of LCN2 with DPN in humans. In this study, serum LCN2 levels were shown to be elevated in individuals with DPN (Fig. 1). Furthermore, multivariable regression analysis showed that serum LCN2 level was independently correlated with the occurrence of DPN in individuals with T2D. Additionally, we found that with the increase in serum LCN2 levels, the TCNS scores for DPN were increased (Fig. 2). Application of TCNS in clinical studies has confirmed its role in documenting and monitoring DPN (14, 21, 22). A higher score indicates more severe disability. Given that LCN2 has been recently shown to be stable in the circulation, this study suggests LCN2 as a biomarker in the evaluation of DPN severity (23).

Coincident with our observations, a most recent study on DPN in mice model has revealed that LCN2 expressions in both DRG and sciatic nerve increase significantly in DPN mice (12). Under the conditions of diabetes, LCN2 from satellite glial cells mediates macrophage infiltration into DRG, stimulates the release of inflammatory cytokines such as tumor necrosis factor-α, and enhances neuronal inflammatory response. PDK2, the key regulator of mitochondrial function, is typically upregulated in diabetic conditions and promotes glycolytic metabolism, along with increased DRG lactic acid production, consequently leading to neurotoxicity. LCN2 contributes to the pathogenesis of DPN via PDK2-lactic axis in DRG of diabetic mice. These findings above may provide mechanic interpretations for our clinical observations.

A variety of factors including renal function, proteinuria, blood glucose, lipid, and BP have been extensively described to influence serum LCN2 levels in individuals with T2D (5, 17, 24, 25). To minimize the potential differences in the comparison of serum LCN2 levels between DPN and NDPN groups, we carefully characterized and matched participants according to sex, age, and BMI. Notably, no statistically significant differences were indicated in other parameters including HbA1c, FBG, FCP, TC, TG, UACR, eGFR, and BP. The only statistically significant difference in the two groups was longer diabetes duration for individuals in the DPN group. This could be explained by the fact that diabetes duration is the risk factor for DPN in T2D (26).

In this study, both BMI and TG were shown to be independent factors associated with serum LCN2 levels, consistent with previous clinical studies (24, 25, 27). Dysregulation of LCN2 has been tied to obesity, metabolic syndrome, and cardiovascular diseases, mainly through its ability to bind to lipids like fatty acids (28). For example, LCN2 could bind to the fatty acid retinoic acid to mediate thermogenesis and lipid metabolism in adipose tissue (29). Additionally, downregulation of LCN2 was shown to attenuate the metabolism of arachidonic acid, impairing energy homeostasis in mice study (30). This study indicated a negative association of LCN2 with DBP, whereas such association was shown to be positive in previous studies (25, 31). This discrepancy may be attributed to the heterogeneity of the studied population across the distinct studies.

A recent study has indicated that a LCN2 MAB significantly reduces cerebral infarction and neurological deficits after stroke, suggesting targeting LCN2 as a promising intervention for the therapy of neurological diseases. Another study showed that treatment with an anti-LCN2 antibody prevented LCN2-related neuroinflammation and neuronal death *in vitro* (20). Therefore, the association of LCN2 with DPN described in this study suggests a presumable strategy for the treatment of DPN.

Limitations should be noted in this study. This was a cross-sectional study, which could not provide the causal relationship between increased serum LCN2 levels and the development and progression of DPN. Moreover, the sample size of this study was limited. Prospective studies with larger sample size are required to unravel the role of LCN2 in the pathogenesis of DPN.

## Conclusion

Taken together, this report is the first study on the association of serum LCN2 with DPN in T2D. LCN2 might be used to evaluate DPN severity. Moreover, LCN2 might serve a role in the pathogenesis of DPN. Novel strategies for the intervention of DPN would be beneficial from further studies on the relationship between LCN2 and DPN.

## Declaration of interest

The authors declare that there is no conflict of interest that could be perceived as prejudicing the impartiality of the research reported.

## Funding

This work was funded by National Natural Science Foundation of China http://dx.doi.org/10.13039/501100001809 (81971264, 81800713), Local Scientific and Technological Development Project Guided by Central Government of China (grant number 2017070802D147), and Integrated Technology Application Research in Public Welfare of Anhui Province (grant number 1704f0804012).

## Author contribution statement

W W conceived and designed this study, wrote this manuscript, and participated in the data analysis. Z X, X R S, and W Z contributed to data collection and analysis. Y X and M Y X contributed to laboratory test and helped in editing the manuscript. S D Y contributed to the conception of this study, and edited the manuscript. S W X, J P W, and Z Z contributed to the study design and critical revision of the manuscript for important intellectual content. All authors read and approved the final version of the manuscript.
